# *In Situ* Natural Product Discovery via an Artificial Marine Sponge

**DOI:** 10.1371/journal.pone.0100474

**Published:** 2014-07-08

**Authors:** James J. La Clair, Steven T. Loveridge, Karen Tenney, Mark O'Neil–Johnson, Eli Chapman, Phillip Crews

**Affiliations:** 1 Xenobe Research Institute, San Diego, California, United States of America; 2 Department of Chemistry and Biochemistry, University of California Santa Cruz, Santa Cruz, California, United States of America; 3 Lead Discovery and Rapid Structure Elucidation Group, Sequoia Sciences, Inc., St. Louis, Missouri, United States of America; 4 Department of Pharmacology and Toxicology, College of Pharmacy, University of Arizona, Tucson, Arizona, United States of America; University of New South Wales, Australia

## Abstract

There is continuing international interest in exploring and developing the therapeutic potential of marine–derived small molecules. Balancing the strategies for ocean based sampling of source organisms versus the potential to endanger fragile ecosystems poses a substantial challenge. In order to mitigate such environmental impacts, we have developed a deployable artificial sponge. This report provides details on its design followed by evidence that it faithfully recapitulates traditional natural product collection protocols. Retrieving this artificial sponge from a tropical ecosystem after deployment for 320 hours afforded three actin–targeting jasplakinolide depsipeptides that had been discovered two decades earlier using traditional sponge specimen collection and isolation procedures. The successful outcome achieved here could reinvigorate marine natural products research, by producing new environmentally innocuous sources of natural products and providing a means to probe the true biosynthetic origins of complex marine–derived scaffolds.

## Introduction

Seminal reviews note that marine ecosystems serve as outstanding sources of molecular structures with activities in antitumor, anti–inflammatory, analgesic, anti–allergy, antiviral, and immunomodulatory screens [1]–[4]. However, recent advances in genomic, proteomic, and microbiological techniques, plus insights from biosynthetic analyses, intimate that many of the key marine–derived therapeutic leads isolated to date such as patellamide [5], the didemnins [6]–[8], the bengamides [9], psymberin [10], or the manzamines [11], [12] may arise from symbiotic microbes and not from the collected organism. In an increasingly appreciated view, while marine–derived small molecules continue to constitute a critical resource in drug discovery, contemporary methods are capable of accessing only a minute fraction of marine diversity. Unfortunately, the majority of marine microbial species have been recalcitrant to cultivation, possibly due to their reliance on complex symbioses [13]. Amazingly, current estimates indicate that over 99% of microbes from environmental samples cannot be cultured under laboratory conditions [14].

While marine–derived small molecules with new modes of action (MOA) are being identified annually [15], the rate of discovery is rather low. A typical natural product program obtains samples by expeditions, where organisms are gathered. The resulting specimens are then shipped back to the laboratory where they are either used as sources for natural product isolation or microbial culturing to obtain compounds. While these methods have produced a number of leads and clinical entities, the procedure is far from being efficient; often many small molecules and microbes are lost [16]. Another drawback is associated with the possible negative environmental impact associated with the large–scale collection of high priority invertebrates including soft corals, sponges, and tunicates [17]. These deficiencies combined with difficulties in correlating leads with clinically relevant MOA [15] and adequate pharmacological properties [16] pose the largest roadblocks to clinical translation.

**Figure 1:**
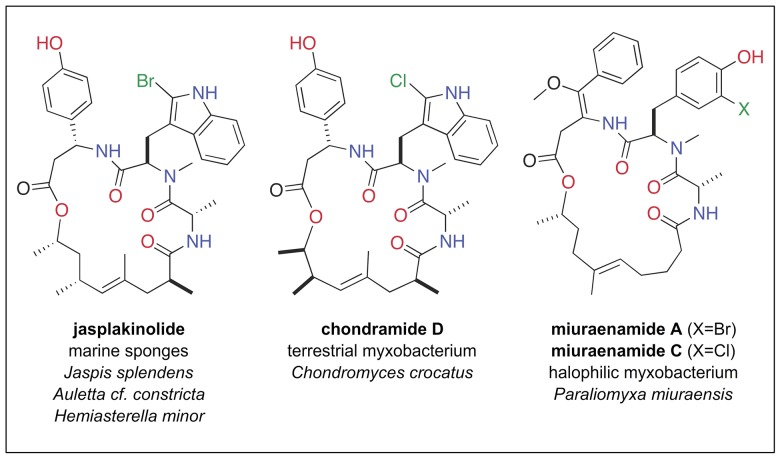
A sponge based hypothesis. A side–by–side comparison of the three natural products shown here illustrates an example of parallel biosynthetic pathways that operated in disparate organisms including marine sponges and myxobacteria. These compounds, arising from the fusion of a triketide with unusual tripeptide moieties, represent the types of biosynthetic products targeted in this study owing to their parallel biogenetic and potential microbial origins. Each compound was previously discovered from the indicated source organisms, and all were subsequently shown to be F–actin stabilizers.

In an effort to circumnavigate such difficulties, we began a campaign to develop an artificial sponge, allowing the first stages of the experimental work to be conducted at sea in an environmentally benign fashion. A guiding principle, inspired in part by prior marine biomimetic robots [18] and deployable isolation systems [19], involved designing a mechanical device that mimics the currently appreciated action of a marine sponge. This device could then serve as a host for microbial communities and as a biological medium for accumulation of secondary metabolites. A proof–of–concept demonstration is presented herein involving the successful use of this artificial sponge to isolate actin–targeting jasplakinolide depsipeptides [20]–[22], a family of natural products that were first identified from sponge hosts collected in the same geographic region as the deployment sites for our artificial sponge (Table 1). It should also be noted that these molecules possess remarkable similarity, as shown in Figure 1, to chondramide D [22], isolated from a terrestrial myxobacterium and the miuraenamides [23], produced by a marine–derived myxobacterium.

**Table 1:** Deployments.

Date	Extract	Location (longitude, latitude)	Depth	T_I_ (h)	T_C_ (d)
05/2011	SES–001	+32°40′11.01″, −117°14′47.04″	35′	5	12
05/2011	SES–002	+32°40′6.46″, −117°14′44.42″	45′	5	12
06/2011	SES–003	+34°0′22.61″, −119°23′28.09″	25′	6	7
06/2011	SES–004	+34°0′22.61″, −119°23′28.09″	35′	6	7
06/2011	SES–005	+34°0′16.85″, −119°25′20.57″	40′	7	7
06/2011	–	+34°0′16.85″, −119°25′20.57″	35′	7	–
06/2011	SES–007	+34°0′16.85″, −119°25′20.57″	25′	7	7
07/2011	SES–008	+1°45′4.28″, +110°30′2.84″	35′	5	14
07/2011	SES-009	+1°45′4.28″, +110°30′2.84″	45′	5	14
07/2011	SES–010	+1°45′4.28″, +110°30′2.84″	25′	5	14
07/2011	SES-011	+1°45′4.28″, +110°30′2.84″	30′	5	14

Date, extract name, location, depth, time of inoculation (T_I_) and time of incubation (T_C_) for the 11 deployments explored in this program. Three expeditions were conducted between the period of 05/02/2011 and 07/31/2011 at three locations. Deployment 6 was lost at sea.

## Materials and Methods

### General

Unless otherwise noted, all reagents and chemical compounds were purchased from Fisher Scientific and used without further purification. NMR samples were dissolved in 13 µL of 100% CD_3_OD (Cambridge Isotope Laboratories), vortexed, and added to a 1 mm tube (Bruker Biospin). ^1^H NMR and ^1^H–^1^H gCOSY NMR spectra were collected on a 600 MHz Avance spectrometer equipped with a 1.7 TCI MicroCryoProbe (Bruker Biospin). FID files were processed using MestReNova version 8.1 (Mestrelab Research) and were referenced relative to the residual solvent peaks. Devices were launched at multiple locations in the Pacific Ocean and the South China Sea at 10–50 m depth. Permits were not required for this deployment. No animal, plant or living matter was collected or damaged during the study nor was biological material collected. The materials were not deployed within protected ecosystems or marine reserves or parks.

### Artificial Sponge

**Figure 2:**
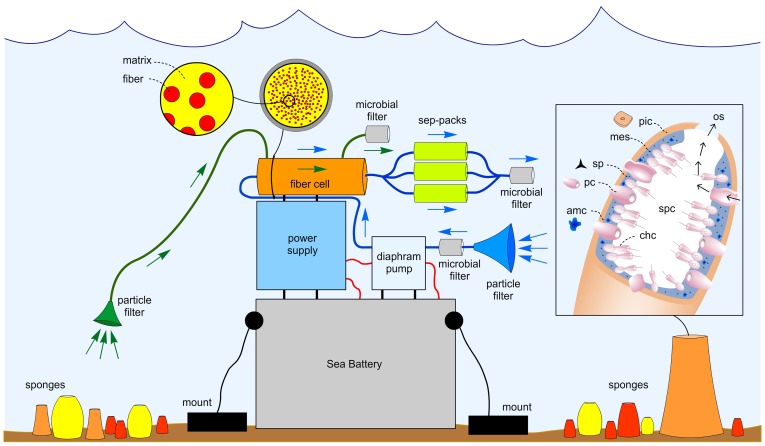
A schematic overview. The artificial sponge was mounted underwater proximal to sponges envisioned to be engaged in secondary metabolite biosynthesis, such as shown in Figure 1. Its components are: particle filters (disposable funnel containing a polyethylene frit, OP–6602–14, ChemGlass), microbial filters (2 µm pore, 50 mm OD, Millex–AP microbial filters, SLA05010, Millipore); a SeaBattery (DeepSea Power & Light); a power supply (self–built); a microdiaphram pump (NF5RP, KNF Neuberger); a hollow–fiber bioreactor (4300–C5011, FiberCell Systems); and parallel–bundle of sep–pak cartridges (ePlastics) containing Amberlite XAD–18 resin (Dow Chemicals). The hollow–fiber bioreactor can act as a bioreactor allowing microbial material to culture inside the artificial sponge. The sep–pak cartridges serve to collect materials either from the seawater or from the microbial content within the hollow–fiber bioreactor. Green arrows indicate flow direction during charging of the hollow–fiber bioreactor from the water column during the inoculation stage (step 1, Figure 3). Blue arrows depict the passage of seawater through the artificial sponge during the incubation stage (step 2, Figure 3). A generic depiction of the anatomy of a sponge is shown within the inset to illustrate the parallel engineered design, codes are: os = osculum, spc = spongocoel, chc = choanocytes, amc = amebocyte, pc = porocytes, sp = spicule, mes = mesohyl, pic = pinacocytes.

**Figure 3:**
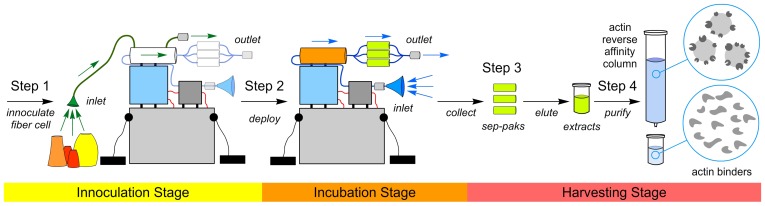
The capture and isolation of natural products through a three–stage, four–step procedure. The process began with inoculation of the hollow–fiber bioreactor (Step 1) followed by incubating the artificial sponge (Step 2) for 7–14 days (Table 1) proximal to the inoculation site. The systems were moved from their inoculation site to a second incubation site, devoid of sponges and fragile sea organisms, in an effort to prevent damage to marine specimens. After the completion, the sep–pak cartridges were harvested and eluted to provide a crude extract (Step 3). A total of 10 extracts were obtained (Table 1). Next, a reverse–affinity method (Step 4) was deployed to isolate natural products based on their ability to bind to actin. This provided a semi–pure fraction from each extract as illustrated in Figure 4 (code: AC–X–A) from extract SES–009 (Table 1). A detailed description of these steps has been provided in the Materials and Methods.

Each artificial sponge was housed within a 2″ ID clear rigid schedule 80 PVC pipe (Harvel) with one of the ends fitted with a cap containing ports for seawater entry, seawater exit, and connections to power. The core of each device was a hollow–fiber bioreactor [24] (C2011, FiberCell Systems), which served to mimic the sponge mesohyl by providing a location to host microbial growth. This bioreactor contained two ports: one for inoculation and a second to pass media during incubation. Seawater was passed through the artificial sponge during both inoculation and incubation using a NF5 RP.51DC–M diaphragm pump (KNF Neuberger), whose flow rate was regulated to pump at 1.2±0.3 L/h through a self–built regulator. Power was provided by an external 6V SeaBattery (DeepSea Power and Light) or a self–built equivalent capable of deployment up to 100 m depth. During incubation, seawater leaving the hollow–fiber bioreactor was passed through three 10 cm ID sep–pak cartridges (ePlastics) containing 50±10 g Amberlite XAD–18 (Dow Chemical Co.) resin/pak, which served as the vehicle for natural product concentration. Components were connected using thick–walled Tygon tubing. Particle filters (OP–6620–14, Chemglass) and 2 µM Millex–AP microbial filters (SLA05010, Millipore) were placed on the inlets and outlets of the artificial sponge to prevent clogging and contamination. A schematic representation of the artificial sponge has been provided in Figure 2 and images of the components are provided in Figure S8 in File S1. The artificial sponges were evaluated during 11 separate deployments (Table 1). Each deployment involved three stages of application (inoculation, incubation, and harvesting) as outlined in Figure 3.

### Inoculation of the Artificial Sponge

Each deployment began by inoculating the hollow–fiber bioreactor, as depicted by Step 1 in Figure 3. Sites were selected that contained multiple species of sponges. Through dive teams, each device was placed such that seawater could be pumped into the inoculation port using a 110 mL disposable funnel containing a polyethylene frit (OP–6602–14, ChemGlass) that was positioned within 1 m of live sponges (typically multiple species of sponges). This funnel served to prevent sediment and particulate matter from entering the artificial sponge. The outlet was capped with a 2 µm 50 mm Millex–AP filter (SLA05010, Millipore) to prevent backflow. After pumping for 5–7 h (Table 1), the inoculation port was closed and the apparatus was moved to a region proximal the sponges used for inoculation (typically within 100 m).

### Incubation of the Artificial Sponge

Once inoculated, each device was incubated for several days proximal to its site of inoculation (Step 2, Figure 3). Each artificial sponge was held in place by anchoring with conventional diving weights and marked by a single buoy at the surface. During this stage, both the inlet and outlet of the artificial sponge were capped with a 2 µm 50 mm Millex–AP filter (SLA05010, Millipore). Routine diving was required to maintain the filters as well as to recharge the battery, the latter was readily achieved by replacement with a charged battery followed by recharging the spent battery. Artificial sponges were operated for 7–14 days, as noted in Table 1.

### Harvesting of the Artificial Sponge

After incubation, the artificial sponge was collected (Step 3, Figure 3). The sep–pak cartridges were removed immediately, washed with deionized H_2_O (3×50 mL), capped, placed in zip lock bags, and stored at 4°C until extraction in a laboratory setting. Extracts were prepared by elution with 95% EtOH (5×50 mL). The fractions were pooled and dried by rotary evaporation in a 250 mL round bottom flask. The material was transferred with EtOH washing to a 20 mL scintillation vial and dried by rotary evaporation followed by lyophilization for 12–18 h, providing extracts SES–001 to SES–011 (Table 1) with 180–630 mg/extract.

### Actin Polymerization Assay

We began by evaluating our extracts for their ability to regulate the polymerization of actin [25] using a commercially available, pyrene labeled assay (BK003, Cytoskeleton, Inc.). The assay was conducted as described in the manufacturer's guidelines. Briefly, purified rabbit muscle actin was modified to contain covalently linked pyrene at the Cys374 residue with 0.6 *N*–1–pyreneacetamide labels per actin monomer. Each assay was conducted with 20 µM pyrene–labeled actin in 5 mM Tris–HCl pH 8.0, 0.2 mM CaCl_2_ and 0.1% DMSO used to solubilize the analyte. Analytes were incubated with the G–actin for 30 min and then polymerization was induced by the addition of 0.2 mM ATP. The assay was run in a final volume of 240 µL in a black 96 well plate (Costar) and monitored using an HTS700 plate reader (Perkin Elmer) with excitation at 360±10 nm and emission at 465±20 nm. Data were collected with a 2.8 s reading time every 34 s. The assay was run four times with three replicates, and each run was obtained with 99.5% of the data points occurring with ≤5% deviation.

### Reverse–Affinity Purification

An aliquot of an Affigel 10 resin (2 mL) bearing 2.2±0.8 mg/mL of rabbit muscle actin (Worthington Biochemical) was incubated with 520 mg of the SES–009 extract in 5 mL of 50 mM phosphate buffered saline (PBS) buffer pH 8.0 containing 0.2 mM CaCl_2_ and 5% DMSO. After shaking by inversion for 12 h at 4°C, the resin was collected via centrifugation on a C–1200 mini–centrifuge (VWR Scientific) for 2 min. The resin was then washed twice with 4 mL 50 mM PBS pH 8.0 containing 0.2 mM CaCl_2_ at 4°C, and once with 4 mL of deionized H_2_O. After each wash the resin was collected by mini–centrifugation. The resulting resin was washed with warm 95% EtOH (2×4 mL). The EtOH fractions were combined and dried via rotary evaporation. The final material was divided into triplicate samples (estimated to be <30 µL total volume) and two samples were used without further purification for NMR and LC–MS analyses, respectively.

### LC–MS Analysis

Ultra high performance liquid chromatography coupled with time–of–flight mass spectrometry (UHPLC/TOF–MS) experiments were performed using an Agilent 1260 binary pump in low dwell volume mode, an Agilent column oven heated to 45°C, and an Agilent 6230 Time of Flight Mass Spectrometer with an electrospray ionization (ESI) source. Experimentally, 1 µL of the AC–X–A sample dissolved in 50% aq. MeOH was injected onto a 1.8 mm particle size, 50×2.3 mm I.D., Zorbax RRHT column. Each sample was subjected to a gradient from 10: 1 to 1: 10 H_2_O: CH_3_CN over 4 min with a 1.5 min hold at 1: 10 H_2_O: CH_3_CN before a 3 min re–equilibration. The flow rate was maintained at 0.8 mL/min. Formic acid, 200 µL/L, was added to both the H_2_O and the CH_3_CN. The TOF–MS was run in positive high–resolution 4 GHz mode with a detector range from 100 *m/z* to 1700 *m/z*. The ESI source was operated with a desolvation temperature of 350°C and a drying gas flow rate of 11 L/min. The fragmentor voltage was held at 135 V while the capillary voltage was ramped from 2500–3000 V.

## Results

The goal to engage in a comprehensive evaluation of the novel artificial marine sponge required completion of several operations. The first step involved building and deploying the device (Figure 3) whose components were designed to mimic the generally understood properties of sponges [26]. This required that the apparatus be robust enough to withstand being placed, for an extended period of time, in the water column proximal an ecosystem rich with sponge biomass. After significant modifications, we settled on the overall design outlined in Figure 2. Ultimately this construct mimicked the general anatomy of a sponge (inset, Figure 2), and required the five primary components shown in Figure 2 and pictorially in Figure S8 in File S1. Briefly, the first module was comprised of a battery, power supply, and a microdiaphragm pump. This assemblage mirrored the function of the sponge aquiferous system (inhalant apertures or pores, inhalant canals, choanocyte chambers, exhalant canals, exhalant apertures or oscules) and provided a uniform flow of seawater at 1.2±0.3 L/h through the artificial sponge, a rate commonly observed in sponges [26]. The next module mimicked the sponge mesohyl, which provided a possible setting to facilitate symbiotic microbial growth. Creating this artificial mesohyl was accomplished by employing a hollow–fiber bioreactor. The idea here was to facilitate the possibility that chemically prolific microbial biota could be captured, maintained, and even flourish within such a matrix. Thus, it seemed essential to constantly bathe the bioreactor contents with nutrients from the water column via pumping seawater through the fibers. The final component, which served a role similar to that of sponge spherulous cells [27], consisted of a parallel bundle of three 10 cm ID sep–pak cartridges containing XAD–18 resin, and was the vehicle for natural product concentration.

Three expeditions were undertaken to test the artificial sponges, two in the Pacific Ocean and one in the South China Sea (Table 1). A total of 11 deployments were conducted using a four–step procedure (Figure 3). Using protocols that were developed by testing the systems in the laboratory (see Materials and Methods), we were able to obtain 10 extracts as given by SES–001 to SES–011, with SES–006 lost at sea (Table 1).

We turned to a commercial actin polymerization assay (see Materials and Methods) as a tool to guide compound isolation. We chose actin polymerization as our primary screen, given the proclivity of marine sponges to contain natural products that are potent modulators of cytoskeleton dynamics, such as the jasplakinolides, the latrunculins, and the swinholides [28]–[29]. To our delight, one of the extracts, coded as SES–009 (Table 1), obtained from placement of the artificial sponge at 45 m depth near Pulau Lakei, Sarawak, Malaysia, displayed an IC_50_≤50 µM (IC_50_ value of 9.2±0.3 µM) in the actin assay.

We then combined this observation with a recently described reverse–affinity (RA) strategy developed in our laboratories [30] to isolate the active principles from extract SES–009 (Figure 3, Step 4). This reverse–affinity procedure (see Materials and Methods) delivered a miniscule sample (<30 µg) given the code AC–X–A (a code that was generated to identify this fraction), which was divided into triplicate samples.

**Figure 4:**
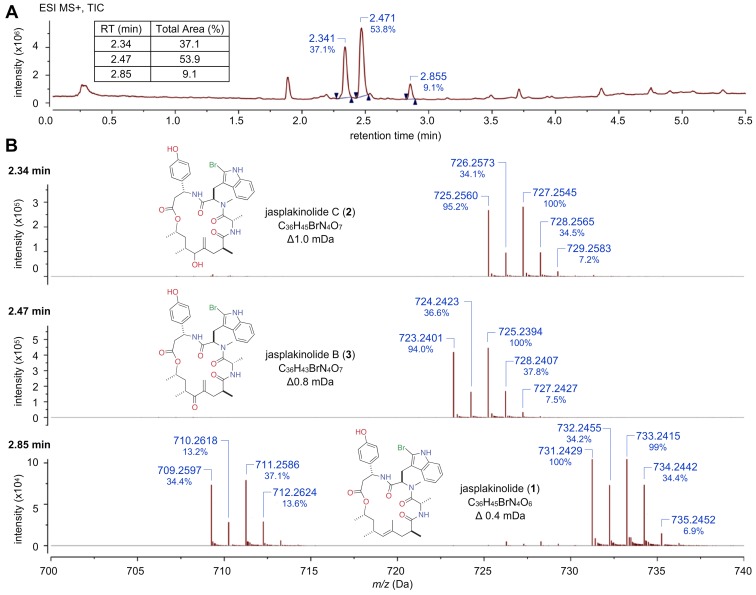
Accurate mass analysis on a <10 µg sample: The actin–bound biosynthetic products were isolated from a sample (code: AC–X–A) via the procedure shown in Figure 3, Step 4. The top panel shows, by Total Ion Chromatogram (TIC) areas, three compounds of interest observed in a ratio of 37∶54∶9 from this material. The three lower panels show AM–MS data for dereplication of compounds at: 2.34 min = jasplakinolide C (**2**), 2.47 min = jasplakinolide B (**3**), and 2.85 min = jasplakinolide (**1**), respectively.

We then turned to modern methods in MS and NMR analysis. LC–MS analysis (see Materials and Methods) of fraction AC–X–A resolved three major peaks in a relative ratio of 4∶6∶1, and each displayed a MS pseudo–molecular ion doublet (Figure 4A) indicating the presence of a bromine atom. The compound with *m/z* = 709/711 was reminiscent of jasplakinolide [20], [21], [31]. Further examination by accurate MS analysis and comparison with potential molecular formulas reported in the literature identified the trio (Figure 4B) as follows: jasplakinolide C (**2**) [27] R_t_ = 2.34 min. *m/z* = 725.2560 [MH^+^] and molecular formula C_36_H_45_BrN_4_O_7_; jasplakinolide B (**3**) [31],[32] R_t_ = 2.47 min. *m/z* = 723.2401 [MH^+^] and molecular formula C_36_H_43_BrN_4_O_7_; and jasplakinolide (**1**) R_t_ = 2.85 min. *m/z* = 709.2597 [MH^+^] and molecular formula C_36_H_45_BrN_4_O_6_.

**Figure 5:**
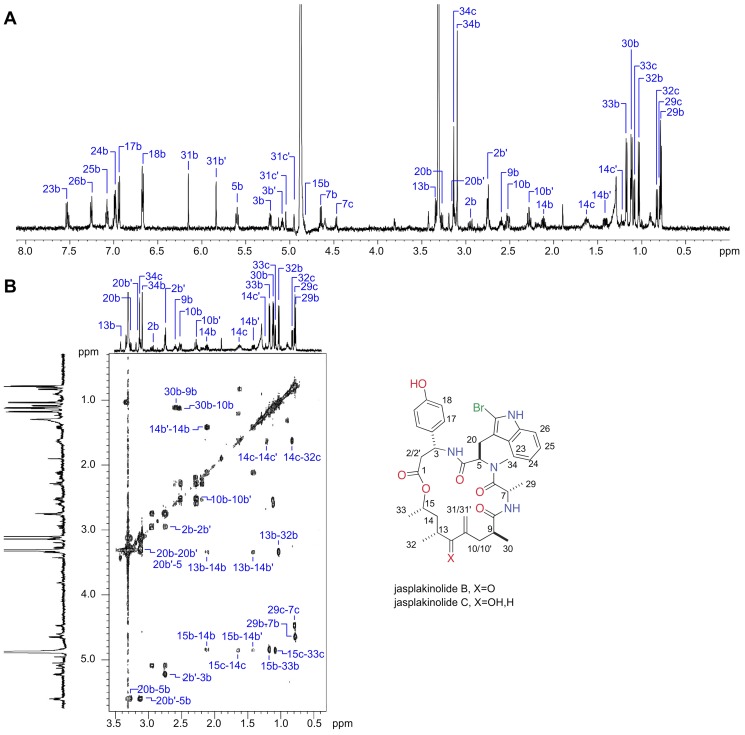
Final dereplication by NMR analysis: Sample AC–X–A (∼10 µg) containing the mixture shown in Figure 4 was subjected to NMR determinations at 26°C in CD_3_OD using a high sensitivity 1.7 mm TCI MicroCryoProbe on a 600 MHz Avance Spectrometer (Bruker Biospin). The annotations shown for the (**A**) ^1^H and (**B**) ^1^H–^1^H gCOSY NMR spectra confirm the dereplication assignments proposed in Figure 4 for jasplakinolide B (**3**) (protons coded as “b”) and jasplakinolide C (**2**) (protons coded as “c”), while resonances for jasplakinolde (**1**) could not be unambiguously observed. Ratios of **3** to **2** were estimated to be 60: 40 by peak areas shown in Figure S7 in File S1.

Analytical data from NMR analyses, including ^1^H NMR (Figure 5A) and ^1^H–^1^H gCOSY NMR spectra (Figure 5B and Figure S2 in File S1) were obtained prior to MS analysis using one of the triplicate samples. Effective data on this microsample could only be obtained using a high sensitivity 1.7 TCI MicroCryoProbe on a 600 MHz Avance (Bruker Biospin) spectrometer (see Figs. S1–S7). Based on the structural hypothesis derived by MS, it was possible to annotate ^1^H NMR resonances for **2** (coded as “c”, Figure 5) and **3** (coded as “b”, Figure 5), while resonances for **1** were either hidden or too small in intensity to be identified. These assignments were additionally verified via side–by–side comparison of the ^1^H NMR data of the mixture to that of natural jasplakinolide B (**3**) (Figure S1 in File S1). Inspection of the ^1^H NMR resonances associated with Me–33, Me–32, and Me–29 (Figure S7 in File S1) indicated that the ratio of jasplakinolide B (**2**): jasplakinolide C (**3**) was 60∶40, in agreement with the ratio derived by MS (Figure 4). Strikingly, this same mixture of three compounds, in a ratio of 18∶1∶1 (compounds **1**: **2**: **3**) was reported from *Jaspis splendens* collected near Vanuatu [32]. Furthermore, as shown in Figure 1, jasplakinolide and its congeners have been reported from three different sponge genera in different taxonomic classes [33]. In addition, the Crews laboratory and others have extensively investigated the structure activity relationships of this class against actin [31], [34]–[36].

## Discussion

One of the eleven deployed artificial sponges (SES–009, Table 1) provided an extract that inhibited actin polymerization. Given each extract was only tested in the actin assay, this is a quite reasonable rate of success. The other extracts likely contain molecules that will display bioactivity to other targets or pathways, and hence the success of this system is not accurately reflected by 1 out of 10. The bioactive fraction was then resolved by reverse–affinity purification. Eventually, a fraction (coded AC–X–A, Figure 3) was discovered to contain a mixture of three members of the jasplakinolide family of depsipeptides, compounds **1**–**3**. Since the discovery of the cyclodepsipeptide, jasplakinolide (**1**), from the sponge *Jaspis splendens* (order Astrophorida, family Ancorinidae) in 1986 by Crews [20] and the Ireland–Faulkner–Clardy consortium [21], sponges in the *Jaspis* (syn: *Dorypleres*) genus have received considerable attention as a source for structurally novel bioactive natural products. Since then, our laboratory has shown that **1** can be found in specimens of *Jaspis* sponges collected throughout the Indo–Pacific Ocean [22], [31]. That we found **1**–**3** in our artificial sponge was in direct agreement with this observation. Importantly, this also represents a critical indicator of the power of such an approach to isolate natural products without having to upset the local ecosystem through invasive collection procedures.

It is relevant to briefly discuss the possible biosynthetic producers or origins of the jasplakinolide mixture isolated from the sep–pak cartridges of the artificial sponge. The only two logical natural sources of the compounds observed here are that: (a) metabolites observed here were those excreted from proximal sponges or microorganisms into the water column and then concentrated in the sep–pak cartridges during the inoculation and/or incubation stages, or (b) metabolites were produced from biosynthetic actions of microorganisms sequestered in the hollow–fiber bioreactor and subsequently collected in the sep–pak cartridges. For the moment, we favor the latter possibility but cannot rule out the former. A confounding issue is that it was impossible to precisely determine the yield of **1**–**3** obtained from the artificial sponges deployed in this study. Two of the triplicate samples of the AC–X–A fraction (<10 µg each), none of which could be accurately weighed, were used in the MS and NMR analyses. It can be assumed that the initial yield from the sep–pak cartridges was undoubtedly greater than the <30 µg obtained. During the incubation stage, the artificial sponge pumped an estimated 380 L of seawater (315 h at 1.2 L/h), at perfect efficiency the seawater column would have to contain ∼0.2 nM of **1**–**3** for this material to be isolated from seawater alone. Alternatively, a microbial consortium would need to operate efficiently to generate the production of **1**–**3** at microgram scales observed in this study. At the present time, the identity of these microbial consortia from each of the bioreactors has not been analyzed. Further experiments are underway to deploy artificial sponges and conduct metagenomic and whole genome sequencing with the goal of unraveling the microbial consortia. This is predicted to be a powerful tool in relating microbiomes to chemical ecology [1]. In addition, these bioreactor data can be compared to the microbiomes of sponge species near the sites of deployment to gain further insight into the origin of these valuable secondary metabolites.

The results presented above delineate an alternative strategy to interrogate a coral reef environment for bioactive chemotypes. This work defines a first–generation deployable artificial marine sponge as a tool to isolate natural products at sea without disturbing the local, fragile ecosystem. We attribute the success realized here to developing an artificial sponge modeled on the anatomy and action of marine sponges. Historically such taxa have served as among the richest sources of natural products, possibly due to the presence of symbiotic colonies of bacteria [37]. However, pinpointing the true source of **1**–**3** concentrated in the sep–pak cartridges constitutes an important and challenging initiative for the future. While our artificial sponge was a first step, these results suggest that further design and optimization of similar marine mechanical artificial devices could provide an ecologically friendly means to access and potentially harvest marine microbes in their natural environment. Overall, this study shows that a mechanical mimic of a sponge provides a non–invasive tool to investigate marine bioorganic chemistry. In addition, with minor modifications the artificial sponges can be tailored to a specific class of molecules. This can be done by using sep–paks with pre–selected biological activity or chemical selectivity [38]. Further molecular diversity may also be obtained from the biological material growing in the fiber cell bioreactor (non–secreted microbial compounds). These artificial sponges also potentially offer a new approach to advance the mining of marine natural product resources and accessing the genomic and metabolomic potential of their associated marine microbes. As mentioned, we have already begun studies to relate microbiomes to chemical ecology using advanced sequencing procedures coupled with state–of–the–art analytical chemistry.

## Supporting Information

File S1 **This file contains Figure S1–Figure S8.** Figure S1, ^1^H NMR stacked plot of sample AC–X–A (bottom: 600 MHz, CD_3_OD) showing jasplakinolide B (protons coded as “b”) and jasplakinolide C (2) (protons coded as “c”) versus natural jasplakinolide B (3) (top: 500 MHz, CDCl3). The jasplakinolide B spectrum was supplied by Prof. D'Aura [32]. This Figure corresponds to Figure 5 providing a direct comparison between the published ^1^H–NMR spectrum of jasplakinolide B (1) (top) with the AC–X–A sample (bottom). Figure S2, Annotated gCOSY ^1^H NMR spectrum of sample AC–X–A (600 MHz, CD_3_OD) with assignments for jasplakinolide B (3) (protons coded as “b”) and jasplakinolide C (2) (protons coded as “c”). This Figure corresponds to Figure 5 providing a copy of the ^1^H–^1^H gCOSY NMR spectrum from the AC–X–A sample used to assign the protons showed in Figure 5. Figure S3, Annotated ^1^H NMR expansion spectrum (0–2 ppm) of sample AC–X–A (600 MHz, CD3OD) with assignments for jasplakinolide B (3) (protons coded as “b”) and jasplakinolide C (2) (protons coded as “c”). This Figure corresponds to Figure 5 offering an expansion of the spectrum from 0–2 ppm. This expansion offers increased resolution of the assigned peaks in the AC–X–A sample. Figure S4, Annotated ^1^H NMR expansion spectrum (2–4 ppm) of sample AC–X–A (600 MHz, CD_3_OD) with assignments for jasplakinolide B (3) (protons coded as “b”) and jasplakinolide C (2) (protons coded as “c”). This Figure corresponds to Figure 5 offering an expansion of the spectrum from 2–4 ppm. This expansion offers increased resolution of the assigned peaks in the AC–X–A sample. Figure S5, Annotated ^1^H NMR expansion spectrum (4–6 ppm) of sample AC–X–A (600 MHz, CD_3_OD) with assignments for jasplakinolide B (3) (protons coded as “b”) and jasplakinolide C (2) (protons coded as “c”). This Figure corresponds to Figure 5 offering an expansion of the spectrum from 4–6 ppm. This expansion offers increased resolution of the assigned peaks in the AC–X–A sample. Figure S6, Annotated ^1^H NMR expansion spectrum (6–8 ppm) of sample AC–X–A (600 MHz, CD_3_OD) with assignments for jasplakinolide B (3) (protons coded as “b”) and jasplakinolide C (2) (protons coded as “c”). This Figure corresponds to Figure 5 offering an expansion of the spectrum from 6–8 ppm. This expansion offers increased resolution of the assigned peaks in the AC–X–A sample. Figure S7, Relative abundance of jasplakinolide B (3) and C (2) in sample AC–X–A (600 MHz, CD_3_OD) as 60: 40, respectively. Annotated ^1^H NMR expansion spectrum (0.7–1.3 ppm) with assignments for jasplakinolide B (3) (protons coded as “B”) and jasplakinolide C (2) (protons coded as “C”). This Figure corresponds to Figure 5 indicating the relative abundance of jasplakinolides B (3) and C (2) in the AC–X–A sample. Figure S8, A close up image of a prototype XRI–S4 depicting the hollow fiber cell culture bioreactor and diaphragm pump. The inoculation and flow ports to the hollow fiber bioreactor are indicated, as well as the flow of seawater through the system (arrows). This Figure corresponds to Figure 2 providing a photograph of the diaphragm pump and fiber cell used within the system (XRI–S4 prototype) applied in these studies.
(PDF)
