# Supplementary material for: In Situ Natural Product Discovery via an Artificial Marine Sponge
Source: PLoS One. 2014 Jul 8;9(7):e100474. doi: 10.1371/journal.pone.0100474 (PMC4086721; doi:10.1371/journal.pone.0100474)
Supplement: File S1 — This file contains Figure S1–Figure S8. Figure S1, 1H NMR stacked plot of sample AC–X–A (bottom: 600 MHz, CD3OD) showing jasplakinolide B (protons coded as “b”) and jasplakinolide C (2) (protons coded as “c”) versus natural jasplakinolide B (3) (top: 500 MHz, CDCl3). The jasplakinolide B spectrum was supplied by Prof. D'Aura [32]. This Figure corresponds to Figure 5 providing a direct comparison between the published 1H–NMR spectrum of jasplakinolide B (1) (top) with the AC–X–A sample (bottom). Figure S2, Annotated gCOSY 1H NMR spectrum of sample AC–X–A (600 MHz, CD3OD) with assignments for jasplakinolide B (3) (protons coded as “b”) and jasplakinolide C (2) (protons coded as “c”). This Figure corresponds to Figure 5 providing a copy of the 1H–1H gCOSY NMR spectrum from the AC–X–A sample used to assign the protons showed in Figure 5. Figure S3, Annotated 1H NMR expansion spectrum (0–2 ppm) of sample AC–X–A (600 MHz, CD3OD) with assignments for jasplakinolide B (3) (protons coded as “b”) and jasplakinolide C (2) (protons coded as “c”). This Figure corresponds to Figure 5 offering an expansion of the spectrum from 0–2 ppm. This expansion offers increased resolution of the assigned peaks in the AC–X–A sample. Figure S4, Annotated 1H NMR expansion spectrum (2–4 ppm) of sample AC–X–A (600 MHz, CD3OD) with assignments for jasplakinolide B (3) (protons coded as “b”) and jasplakinolide C (2) (protons coded as “c”). This Figure corresponds to Figure 5 offering an expansion of the spectrum from 2–4 ppm. This expansion offers increased resolution of the assigned peaks in the AC–X–A sample. Figure S5, Annotated 1H NMR expansion spectrum (4–6 ppm) of sample AC–X–A (600 MHz, CD3OD) with assignments for jasplakinolide B (3) (protons coded as “b”) and jasplakinolide C (2) (protons coded as “c”). This Figure corresponds to Figure 5 offering an expansion of the spectrum from 4–6 ppm. This expansion offers increased resolution of the assigned peaks in the AC–X–A sample. Figure S6, Ann [file pone.0100474.s001.pdf]

SUPPORTING INFORMATION

In Situ Natural Product Discovery via an Artificial Marine Sponge

James J. La Clair,[1,*] Steven T. Loveridge,[2] Karen Tenney,[2] Mark O'Neil–Johnson,[3] Eli Chapman,[4,*] and Phillip Crews[2,*]

[1]Xenobe Research Institute, P. O. Box 3052, San Diego, CA 92164, USA.

[2]Department of Chemistry and Biochemistry, University of California, Santa Cruz, Santa Cruz, CA 95064, USA.

[3]Lead Discovery and Rapid Structure Elucidation Group, Sequoia Sciences, Inc., 1912 Innerbelt Business Center Drive, St. Louis, MO 63114, USA.

[4]College of Pharmacy, Department of Pharmacology & Toxicology, University of Arizona, 1703 E. Mabel St., P. O. Box 210207, Tucson, AZ 85721–0207, USA.

*To whom correspondence should be addressed. E–mail: pcrews@ucsc.edu (P. C.); i@xenobe.org (J. J. L.); chapman@pharmacy.arizona.edu (E. C.).

Supplementary Materials:

Figures S1 – S8

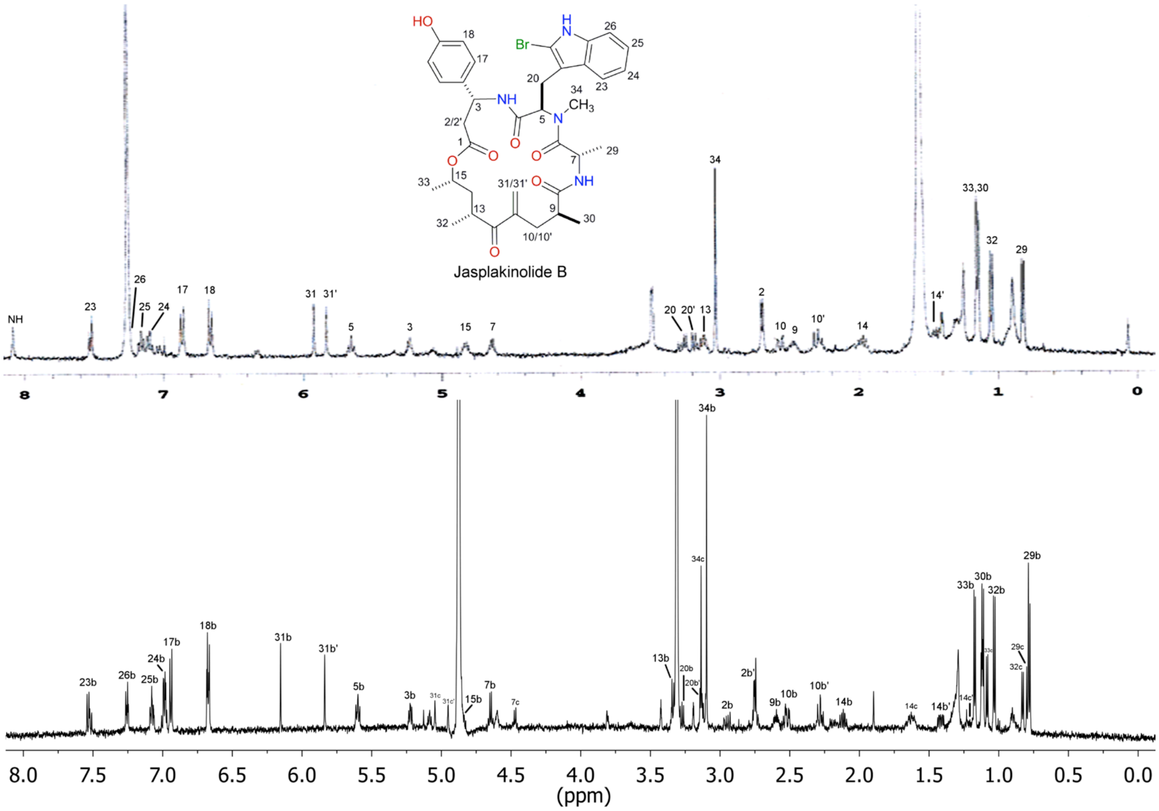

Fig. S1. ^{1}H NMR stacked plot of sample AC–X–A (bottom: 600 MHz, CD_3OD) showing jasplakinolide B (protons coded as "b") and jasplakinolide C (**2**) (protons coded as "c") versus natural jasplakinolide B (**3**) (top: 500 MHz, $CDCl_3$). The jasplakinolide B spectrum was supplied by Prof. D'Aura [*32*].

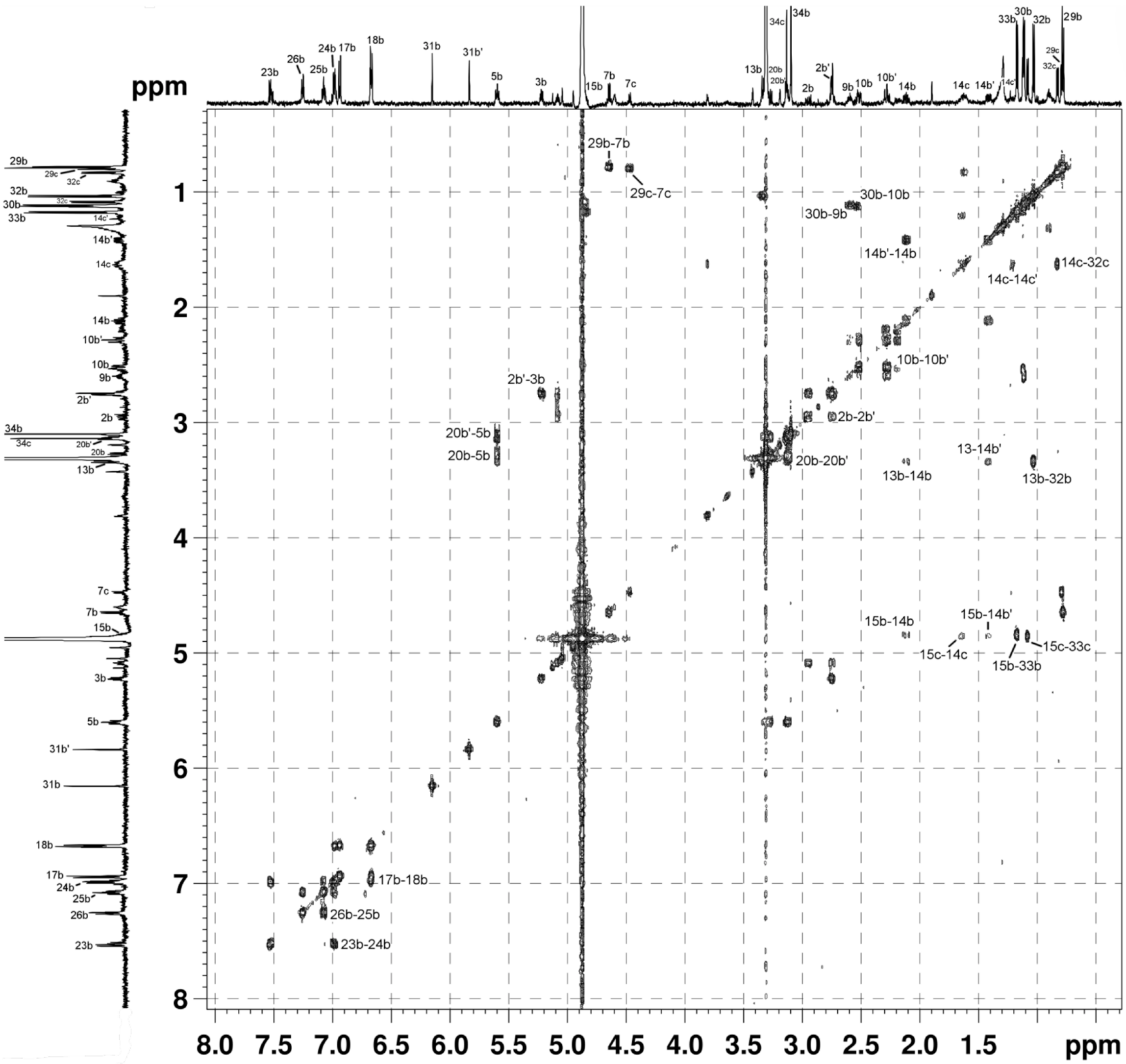

Fig. S2. Annotated ^{1}H–^{1}H gCOSY NMR spectrum of sample AC–X–A (600 MHz, CD_3OD) with assignments for jasplakinolide B (**3**) (protons coded as "b") and jasplakinolide C (**2**) (protons coded as "c").

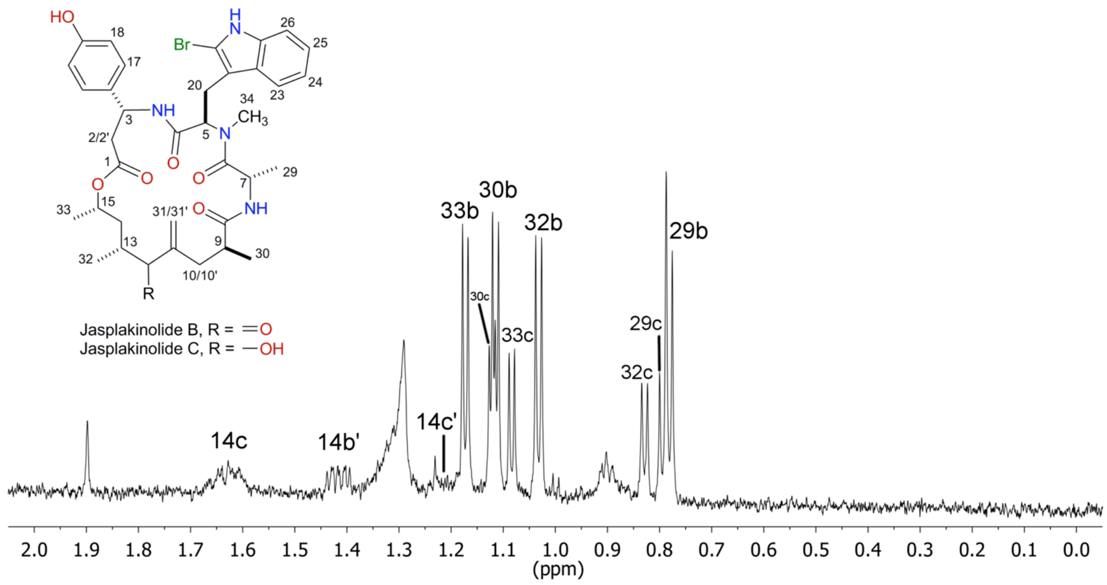

Fig. S3. Annotated ^{1}H NMR expansion spectrum (0–2 ppm) of sample AC–X–A (600 MHz, CD_3OD) with assignments for jasplakinolide B (**3**) (protons coded as "b") and jasplakinolide C (**2**) (protons coded as "c").

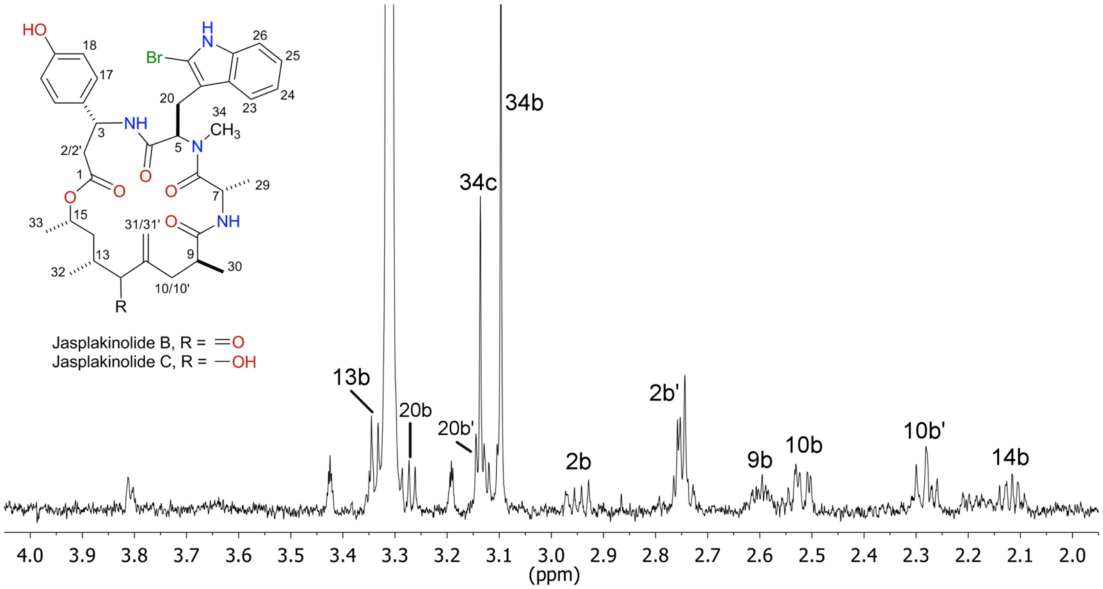

Fig. S4. Annotated ^{1}H NMR expansion spectrum (2–4 ppm) of sample AC–X–A (600 MHz, CD_3OD) with assignments for jasplakinolide B (**3**) (protons coded as "b") and jasplakinolide C (**2**) (protons coded as "c").

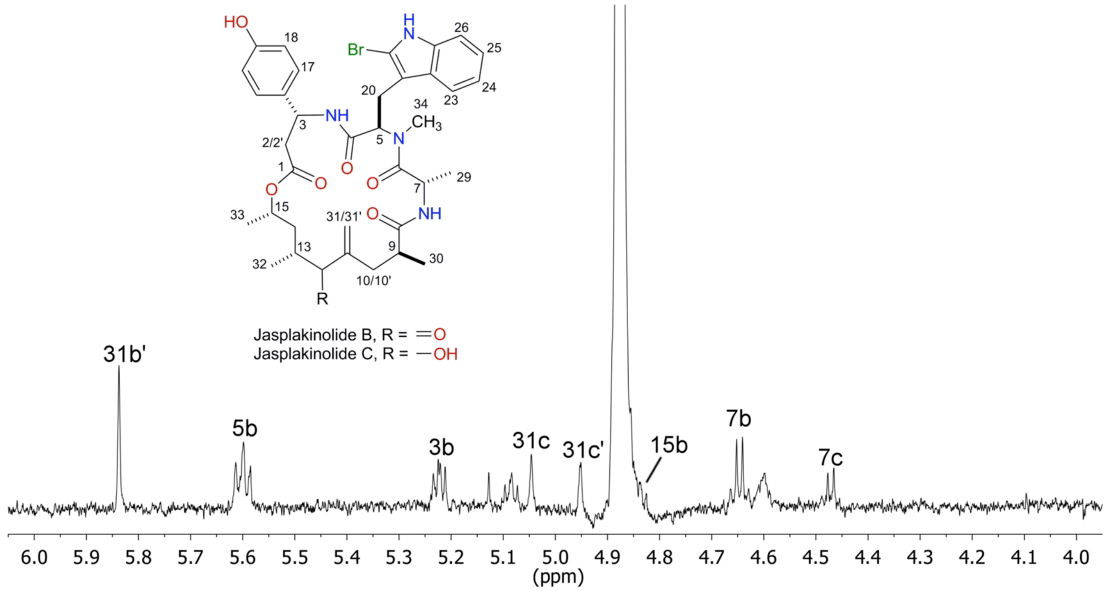

Fig. S5. Annotated ^{1}H NMR expansion spectrum (4–6 ppm) of sample AC–X–A (600 MHz, CD_3OD) with assignments for jasplakinolide B (**3**) (protons coded as "b") and jasplakinolide C (**2**) (protons coded as "c").

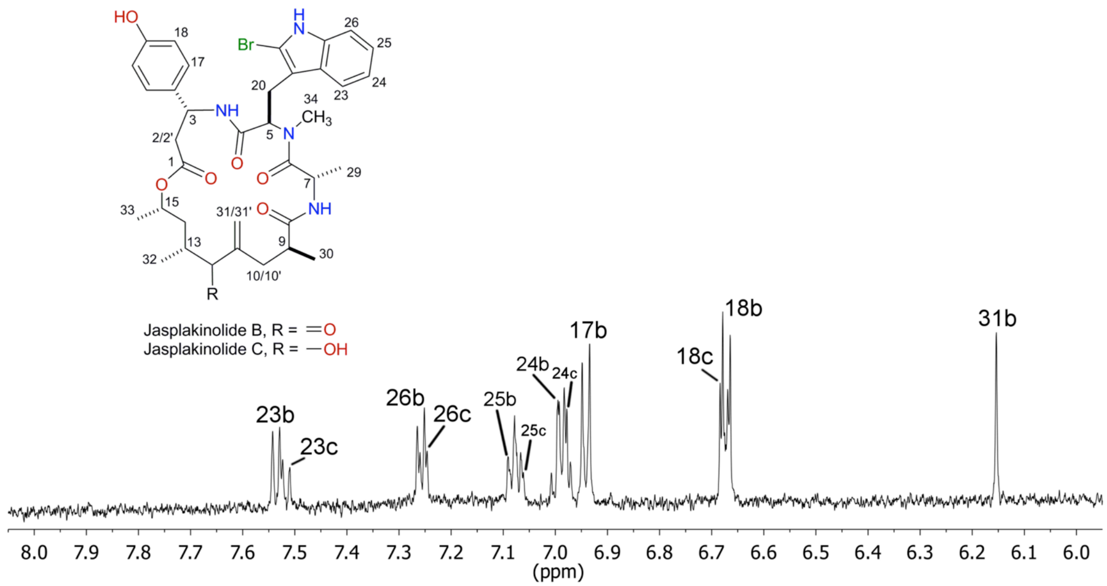

Fig. S6. Annotated ^{1}H NMR expansion spectrum (6–8 ppm) of sample AC–X–A (600 MHz, CD_3OD) with assignments for jasplakinolide B (**3**) (protons coded as "b") and jasplakinolide C (**2**) (protons coded as "c").

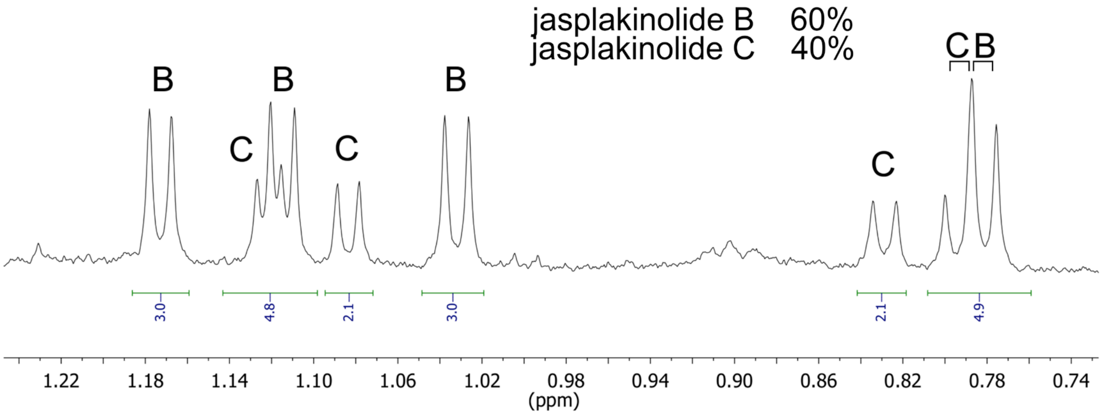

Fig. S7. Relative abundance of jasplakinolide B (**3**) and C (**2**) in sample AC–X–A (600 MHz, CD_3OD) as 60:40, respectively. Annotated ^1H NMR expansion spectrum (0.7 –1.3 ppm) with assignments for jasplakinolide B (**3**) (protons coded as "B") and jasplakinolide C (**2**) (protons coded as "C").

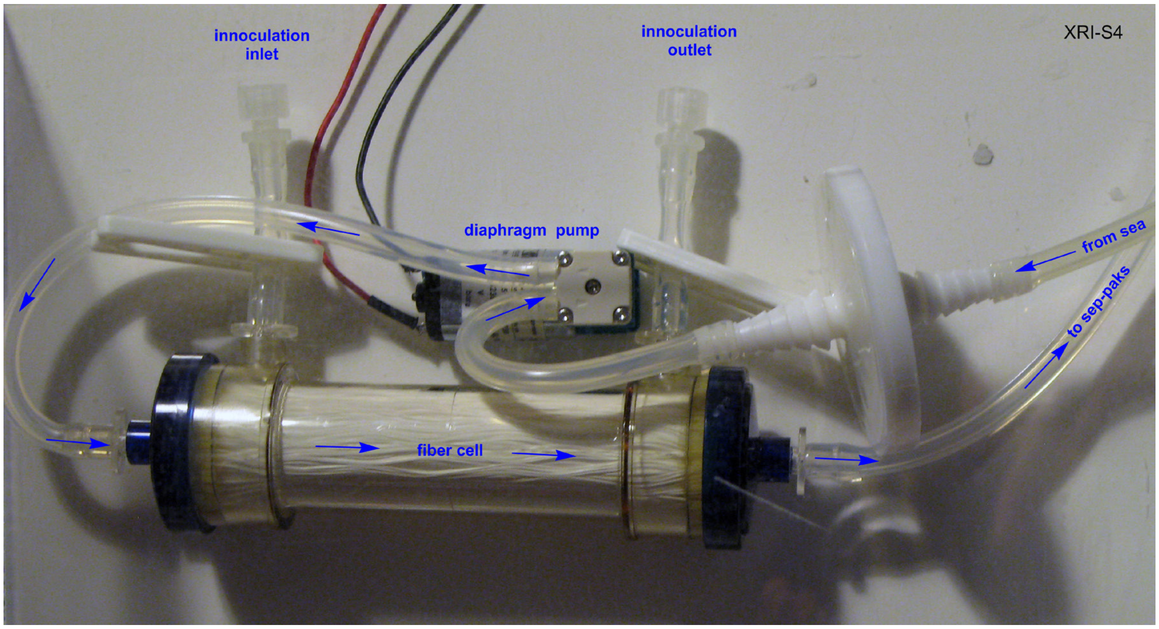

Fig. S8. A close up image of a prototype XRI–S4 depicting the hollow fiber cell culture bioreactor and diaphragm pump. The inoculation and flow ports to the hollow fiber bioreactor are indicated, as well as the flow of seawater through the system (arrows).
